# The Impact of Clinical and Cognitive Variables on Social Functioning in Parkinson's Disease: Patient versus Examiner Estimates

**DOI:** 10.4061/2010/263083

**Published:** 2010-03-11

**Authors:** Patrick McNamara, Karina Stavitsky, Raymon Durso, Erica Harris

**Affiliations:** ^1^Department of Neurology, Boston University School of Medicine and VA New England Healthcare System, 72 East Concord Street, B528, Boston, MA 02118, USA; ^2^Department of Psychology, Boston University, Boston, MA 02215, USA

## Abstract

*Purpose*. To assess the impact of clinical variables on social skills and behaviors in Parkinson's disease (PD) patients and patient versus examiner estimates of social functioning. *Methods*. Twenty-eight patients with PD and 32 controls with chronic disease were assessed with a battery of neuropsychologic, personality, mood, and social function tests. *Results*. Patients' estimates of their own social functioning were not significantly different from examiners' estimates. The impact of clinical variables on social functioning in PD revealed depression to be the strongest association of social functioning in PD on both the patient and the examiner version of the Social Adaptation Self-Evaluation Scale. *Conclusions*. PD patients appear to be well aware of their social strengths and weaknesses. Depression and motor symptom severity are significant predictors of both self- and examiner reported social functioning in patients with PD. Assessment and treatment of depression in patients with PD may improve social functioning and overall quality of life.

## 1. Introduction

A series of recent studies suggest that patients with Parkinson's disease (PD) present with impairments in social behaviors and social functioning [1–4]. Changes in the quality of social interactions, furthermore, may predate onset of overt extrapyramidal motor signs of PD by several years [5, 6]. Thus, a better understanding of the determinants of social functioning in PD is needed.

Deficits in executive cognitive functions that are often associated with PD [7] may also influence social functioning and self-monitoring of social behaviors. Specific personality and behavioral changes are often observed in patients with deficits in frontal lobe functioning. These personality changes include problems with behavioral disinhibition, apathy, disinterest, interpersonal communication, and irritability [1, 8–11]. Patients who present with executive deficits and personality changes associated with these deficits would likely also evidence impairments in social functioning such as those related to social interactions and empathetic responding.

Mood and general cognitive function may also be factors that influence social functioning. For example, patients who are depressed often show impaired social behaviors [12] and show improvements in a variety of social domains after treatment with antidepressant medication [13]. It is also likely that clinical factors such as disease severity, mood and cognitive dysfunction will negatively impact social functioning in PD.

To date, no studies have simultaneously assessed effects of patients' own awareness of social functioning or of the impact of clinical, cognitive and personality variables on social functioning in PD. In the following study, we evaluated social functioning in PD by simultaneously assessing the contribution of several clinical variables to both self-reported and examiner-rated social behaviors (including social functioning and empathy) in PD.

## 2. Methods

The study was approved by the Veterans Affairs (VA) Internal Review Board for protection of human subjects. One of the authors (Erica Harris) presented each participant with a detailed informed consent agreement that described the risks and procedures of the study. After the participant indicated consent and signed the form, testing began.

### 2.1. Participants

Twenty-eight nondemented patients with PD (2 female) were recruited from the outpatient Movement Disorders Clinic at the VA Boston Healthcare System, Boston, MA. Patients were individually diagnosed by Dr. Raymon Durso, director of the clinic. All were right-handed. Ten patients were at Hoehn-Yahr (H-Y) Stage II and 15 at Stage III. All patients were rated for disease severity on the Unified Parkinson's Disease Rating Scales (UPDRS) by a PD specialist (Raymon Durso). All participants scored 25 and above on the Mini-Mental State Examination [14] and none were demented according to clinical examinations and DSM-IV [15] criteria. All were on some form of dopaminergic medication and were tested while on medications with optimal effects (i.e., motor signs were well controlled). Medication information was obtained from each patient by the neurologist and levodopa equivalent dosages were calculated based on previous reports with 100 mg levodopa = 83 mg levodopa with a COMT inhibitor = 1 mg pramipexole = 1 mg pergolide [16]. Thirty-two control subjects (CS; 12 female) were also recruited from the VA community. All of these control participants had some form of chronic disease or medical complaint such as low back, head or neck chronic pain syndromes. All participants with a history of substance abuse or head injury were excluded.

## 3. Measures

### 3.1. Mood Tests

We assessed depression, stress and anxiety with the short-form of the Depression Anxiety Stress Scales (DASS) developed by S.H. Lovibond and P.F. Lovibond [17]. Crawford and Henry [18] and Antony et al. [19] have reported excellent reliability, validity and other psychometric properties for the three subscales of the DASS. The test includes 21 questions, 7 in each of the depression, anxiety and stress subscales with higher scores indicating greater impairment. The DASS Manual [17] reports the following cutoff scores for depression (normal = 0–9, mild = 10–13, moderate = 14–20, severe = 21–27, and extremely severe = 28+); anxiety (normal = 0–7, mild = 8–9, moderate = 10–14, severe = 15–19, and extremely severe = 20+), and stress (normal = 0–14, mild = 15–18, moderate = 19–25, severe = 26–33, and extremely severe = 34+). The DASS Manual indicates that the scores of the DASS_21_ are comparable to the scores of the full length DASS but that the scores will need to be doubled before comparison to the cutoff scores. The DASS Manual also reports Cronbach's alpha-coefficients for each of the 3 subscales: depression = 0.81, Anxiety = 0.73 and Stress = 0.81 [17].

### 3.2. Stroop Color-Word Interference Procedure

To assess executive cognitive functioning, we used the Stroop color-word interference test. This test requires the subject to name the color of the ink or to name the word of a color-word that is printed [20]. An “interference” test card consists of rows of color words printed in ink colors incongruent with the word represented, with the task being to name the ink colors as quickly as possible. Susceptibility to cognitive interference is calculated as the total time taken to name the colors and read the words. PET studies show that orbitofrontal cortex is activated in healthy subjects during the interference condition [21].

### 3.3. Social Adaptation Self-Evaluation Scale (SASS)

To quantitatively assess quantity and quality of overt social behaviors, we used the Social Adaptation Self-Evaluation Scale (SASS). The SASS [13] is a 21-item questionnaire that is administered to patients (SASS-P) to obtain self-ratings about behavior and motivation. A virtually identical version of the questionnaire is provided to the examiner or to a significant other (SASS-E) to fill out with respect to the patient. The items target specific types of social behavior. We required the rater to rate the quality of those behaviors and if they occur at all. “Quality” refers to how well the social behavior is adapted to social context and how well the behavior accomplishes intended goals of the patient. Ratings for each social behavior range from 0–3 (0 = no, 1 = minimal, 2 = medium, and 3 = maximal social adjustment). The minimum score for both the self-report and examiner versions of the scale is 0 and the maximum score is 60. Higher scores indicate greater adaptation to the social environment. Using this scale, “normal” social functioning was determined to be a score of 35–52; therefore, impaired functioning is any score below 35. The SASS evidences good face validity, internal and external validity, test-retest reliability, and a Cronbach's alpha-coefficient of 0.74 for all individual items [13].

### 3.4. Empathy Quotient

 The ability to empathize with another is a core social ability as it allows one to take the perspective of another in any given social exchange. To assess interpersonal empathy ability, we administered an empathy scale devised by Baron-Cohen and Wheelwright, The Cambridge Behaviour Scale [22]. It contains a total of 60 self-report statements with only 40 of those statements tapping empathy and the remaining 20 statements acting as fillers. About half of the statements were worded to produce disagree responses so that biased responding sets could be checked. The empathy scale yields an “empathy quotient”, or EQ. The minimum EQ score is 0 and the maximum score is 80, with one point given if the empathic statement is endorsed mildly (slightly agree) or two points given it if is endorsed strongly (strongly agree). The same scoring rules applied to those empathic statements that elicited a “strongly disagree” or “slightly disagree” responses [23]. Test-retest reliability for the EQ is *r* = 0.97 [22]. 

## 4. Statistical Analyses

### 4.1. The Social Functioning Data in Both the CS and the PD Participants Was Normally Distributed

Independent student's t-tests were performed to compare differences between CS and PD groups on all demographic and clinical variables as well as variables related to social functioning. Paired-samples t-tests were performed in order to examine differences between patient and examiner report of social functioning in both PD and CS groups. A multiple regression analysis was performed to examine which clinical and cognitive variables significantly accounted for variance in patient and examiner ratings of social functioning as well as interpersonal empathy. The analyses were performed using DASS-anxiety, DASS-depression, age, disease stage, disease duration, UPDRS total, MMSE and Stroop interference as predictors and SASS-P, SASS-E, and EQ as the criterion variables.

## 5. Results

Demographic and clinical differences between PD and CS participants are presented in Table 1. PD participants were significantly older, had lower MMSE scores and had higher DASS total scores. We therefore treated these latter three variables (age, MMSE score and mood) as covariates in all the analyses that follow.

### 5.1. Overall Differences in Social Functioning and Empathy in PDs versus Controls

No significant differences in social functioning or empathy score were found between PD and CS participants, even when controlling for age, MMSE and DASS total mood score (see Table 2). 

### 5.2. Examiner versus Patient's Ratings on the SASS

There were no significant differences on any of the individual SASS items or on the total scores between examiner and self-report of social functioning in either PD or CS groups.

### 5.3. Predictors of Examiner Estimates on Patient Social Functioning

The next set of analyses was performed to examine the relationship of clinical variables to examiner's estimates of patient social functioning (see Table 3). The overall model was significant accounting for 73% of variance in SASS-E score. When each variable was examined, DASS-depression was the only significant predictor of social functioning in patients with PD as reported by the observers (*β* = −.67, *P* = .002). 

### 5.4. Predictors of Patient's Self-Rankings on Social Behaviors

 This analysis was repeated for the patient version of the SASS and again the overall model was significant accounting for 75% of variance in SASS-P score (Table 3). For this version of the SASS, DASS-depression was the strongest predictor of patient self-rating of their social functioning (*β* = −.60, *P* = .004) followed by UPDRS total (*β* = −.44, *P* = .04).

### 5.5. Predictors of Interpersonal Empathy

A third set of multivariate regression analyses was performed to examine the impact of the same set of clinical variables (DASS-anxiety, DASS-depression, age, disease stage, disease duration, UPDRS total, MMSE, and Stroop interference) on the total score on the EQ (see Table 3). These analyses revealed that none of the above variables were significant predictors of the EQ score.

## 6. Discussion

In the current study, we found that patients with PD and close examiners of these individuals did not differ in rating their social behaviors. PD patients appear to be well aware of their social strengths and weaknesses. A multivariate analysis of the impact of clinical variables on social functioning in PD revealed that depression is the strongest predictor of social functioning in PD for examiner and patient-rated scores of social functioning. In addition, an analysis of patients' self-rating of their own social interactions also revealed that motor symptom severity may impact social functioning in patients with PD. None of the clinical or cognitive variables were significant predictors of empathy in those with PD. These findings suggest that patients estimate their social interaction abilities on the basis of their own experience of disease severity and their overall mood while close examiners rely on observed mood states of the person with PD when judging the quality of PD social interactions. 

Social functioning requires communication of ideas and emotions between persons involved in a social interaction. Communication of emotion between people requires an ability to empathize with others as well as effective conveyance of visual impressions, facial displays, speech prosody patterns and gesture, among other capacities. Our data suggest that the ability to empathize with others appears to be intact in persons with PD. Nevertheless, independent lines of evidence [24–26] suggest that PD patients exhibit impaired recognition of emotional expression in the faces of other people. This impairment persists even after controlling for visual discrimination deficits. Other symptoms of PD such as masked facies and dysarthria can make communication difficult and thus influence the quality and quantity of social interactions. Crucian et al. [27] reported induction of verbal kinesia paradoxica in some patients with PD who were asked to recall emotional episodes. Patients could not adequately express their memory-linked emotions. All of these facts suggest that PD patients, particularly depressed PD patients, may be impaired in the expression and recognition of social emotions. 

Patients with primary depression (without PD) have also been reported to be impaired on a variety of social domains [12]. In these patients, treatment of depression is linked to improvement in social functioning [13]. In the current study, we found that in patients with PD, depression was also linked to greater impairment in social functioning. Almost half of PD patients (45%) experience clinically significant levels of depression [28]. Depression in PD is not related to the motor symptom severity or the duration of the disease [28]. However, it significantly reduces quality of life, and as we document here, it impairs social interaction and social behaviors.

Our study has limitations. We did not assess apathy which is a common nonmotor symptom of PD that is associated with reduced motivation and reduced initiation of social interactions. Thus, we cannot rule out the possibility that some of the patients had apathy and that apathy, in addition to mood and motor deficits, was influencing social outcomes. We also used a small convenience sample, and therefore, our participants may not be representative of the larger PD population. PD is heterogeneous in its presentation and a small sample may not sufficiently represent the various symptom profiles of patients in the general population. Because of its heterogeneity, various studies have aimed to investigate meaningful subtypes of the disease, an effort that is important for understanding the etiology of the disorder and disease management. Due to the sample size limitation and lack of power we were not able to analyze subgroups of patients, which would give a more thorough examination of the association of depression and social functioning. A larger scale study is therefore needed to investigate this issue more thoroughly. The current study did not include any diagnostic measures of depression so we were not able to examine whether patients who were clinically depressed presented with deficits in social functioning. However, our study demonstrates that higher scores on a measure of depression do significantly predict lower scores on a measure of social functioning. Future research is needed to more thoroughly investigate the contribution of clinically diagnosed depression to social functioning in PD. In addition, we did not measure all relevant variables for an analysis of social functioning in PD. A key aspect of social cognition is the ability to infer other peoples' mental states, thoughts and feelings. This is referred to as Theory of Mind (ToM), or mentalizing ability. We did not measure ToM abilities in our participants so we could not assess the potential impact of ToM performance on social skills in PD. To our knowledge, only two studies have been published on ToM abilities in PD [4, 29]. Both studies reported that PD patients were impaired on at least one ToM task.

In summary, this study suggests that both the awareness of and social dysfunction itself in PD may be a function of various disease-related factors such as depression and motor symptom severity. Treating the depression may improve social functioning and improve quality of life in these patients. Various studies show that pramipexole improves depression in addition to motor symptoms in patients with PD [28]. By studying the social cognitive abilities of patients and the relation of these to other clinical characteristics of the disease, we have for the first time quantitatively described elements of social interaction in PD in relation to well-defined clinical factors, an area previously neglected in earlier studies of behavioral impairment in PD.

## Figures and Tables

**Table 1:** Comparison of clinical and demographic variables in CS and PD participants.

	CS (*n* = 32)	PD (*n* = 28)	Significance
Age	56.3 (9.2)	66.5 (11.9)	*P* < .0001***
Education	15.3 (2.7)	13.9 (3.0)	ns
MMSE	28.3 (1.5)	26.6 (1.7)	*P* < .0001***
DASS—Anxiety	2.12 (2.4)	5.32 (4.1)	*P* < .0001***
DASS—Depression	3.94 (4.7)	4.32 (4.3)	ns
Stroop 3 Interference	46.8 (29.8)	51.3 (29.8)	ns
Disease Duration	N/A	9.01 (4.96)	N/A
H and Y Stage^a^	N/A	3 (2–3)	N/A
UPDRS total	N/A	25.3 (13.1)	N/A
LDE	N/A	628.9 (330.8)	N/A

Notes: ***P < .0001; ns: not significant; N/A: not applicable; CS: control; PD: Parkinson's disease; MMSE: mini-mental state exam; DASS: Depression Anxiety Stress Scales; H & Y Stage: Hoehn & Yahr Stage; UPDRS: Unified Parkinson's Disease Rating Scale; LDE: Levodopa Dosage Equivalent.

^a^Median (range) is reported.

**Table 2:** Comparison of CS and PD participants on SASS and EQ variables.

	CS (*n* = 32)	PD (*n* = 28)	Significance
SASS-P total	42.8 (10.7)	40.4 (7.6)	ns
SASS-E total	44.6 (11.1)	41.4 (9.7)	ns
EQ Total	40.7 (12.7)	39.3 (12.3)	ns

Notes: ns: not significant; CS: control; PD: Parkinson's disease; SASS-P: Social Adaptation Self-Evaluation Scale, Patient version; SASS-E: Social Adaptation Self-Evaluation Scale, Examiner version; EQ: The Cambridge Behaviour Scale, empathy quotient.

**Table 3:** Results of multiple regression analyses of clinical variables as predictors of social functioning in patients with PD. Values presented are the standardized coefficient Beta (*β*) and *P*-values for SASS-E, SASS-P, and EQ.

	*β* (*P* value)		
	SASS-E	SASS-P	EQ
Disease Duration	−.31 ± .28 (*P* = .06)	.16 ± .22 (*P* = .34)	−.33 ± .48 (*P* = .13)
Disease Stage	−.06 ± .59 (*P* = .66)	−.12 ± .46 (*P* = .43)	−.16 ± .83 (*P* = .42)
UPDRS Total	−.27 ± .14 (*P* = .18)	−.44 ± .11 (*P*=.04)*	−.38 ± .25 (*P* = .15)
MMSE	.32 ± 1.56 (*P* = .10)	−.30 ± 1.23 (*P* = .14)	−.30 ± 2.66 (*P* = .25)
Age	−.04 ± .13 (*P* = .79)	−.09 ± .10 (*P* = .60)	.11 ± .22 (*P* = .62)
DASS Anxiety	−.11 ± .46 (*P* = .56)	−.16 ± .37 (*P* = .41)	−.12 ± .79 (*P* = .65)
DASS Depression	−.67 ± .42 (*P*=.002)**	−.60 ± .33 (*P*=.004)**	−.28 ± .71 (*P* = .27)
Stroop Interference	.14 ± .07 (*P* = .52)	−.08 ± .05 (*P* = .71)	−.54 ± .12 (*P* = .08)

Notes: *P < .05; **P < .01; SASS-E: Social Adaptation Self-Evaluation Scale, Examiner version; SASS-P: Social Adaptation Self-Evaluation Scale, Patient version; EQ: The Cambridge Behaviour Scale, empathy quotient; MMSE: mini-mental state exam; DASS: Depression Anxiety Stress Scale; UPDRS: Unified Parkinson's Disease Rating Scale.
